# Glycolysis- and immune-related novel prognostic biomarkers of Ewing's sarcoma: glucuronic acid epimerase and triosephosphate isomerase 1

**DOI:** 10.18632/aging.203242

**Published:** 2021-07-07

**Authors:** Jie Jiang, Xinli Zhan, Guoyong Xu, Tuo Liang, Chaojie Yu, Shian Liao, Liyi Chen, Shengsheng Huang, Xuhua Sun, Ming Yi, Zide Zhang, Yuanlin Yao, Chong Liu

**Affiliations:** 1 The First Clinical Affiliated Hospital of Guangxi Medical University, Nanning, Guangxi Province, China

**Keywords:** Ewing's sarcoma, immunohistochemistry, glycolysis, immune infiltration, WGCNA

## Abstract

Introduction: Owing to the poor prognosis of Ewing's sarcoma, reliable prognostic biomarkers are highly warranted for clinical diagnosis of the disease.

Materials and Methods: A combination of the weighted correlation network analysis and differentially expression analysis was used for initial screening; glycolysis-related genes were extracted and subjected to univariate Cox, LASSO regression, and multivariate Cox analyses to construct prognostic models. The immune cell composition of each sample was analysed using CIBERSORT software. Immunohistochemical analysis was performed for assessing the differential expression of modelled genes in Ewing's sarcoma and paraneoplastic tissues.

Results: A logistic regression model constructed for the prognosis of Ewing's sarcoma exhibited that the patient survival rate in the high-risk group is much lower than in the low-risk group. CIBERSORT analysis exhibited a strong correlation of Ewing's sarcoma with naïve B cells, CD8^+^ T cells, activated NK cells, and M0 macrophages (P < 0.05). Immunohistochemical analysis confirmed the study findings.

Conclusions: *GLCE* and *TPI1* can be used as prognostic biomarkers to predict the prognosis of Ewing's sarcoma, and a close association of Ewing's sarcoma with naïve B cells, CD8^+^ T cells, activated NK cells, and M0 macrophages provides a novel approach to the disease immunotherapy.

## INTRODUCTION

Ewing's sarcoma, the second most frequent orthopaedic malignancy, is a highly aggressive, low-grade, small, round, and blue-cell tumour, with undefined pathogenesis [1, 2]. It can develop at any age; however, its incidence particularly in young people is high. Current treatment modalities only seek to control its metastasis through a combination of neoadjuvant and adjuvant chemotherapy, as well as surgery and radiation therapy, although the prognosis of patients with recurrence remains poor [3]. Studies have demonstrated that the prognosis of Ewing's sarcoma is largely dependent on the occurrence of metastases, and the survival rate is reduced to approximately 20%–30% in case of metastasis [4]. A study on Ewing's sarcoma of the head and neck reported that the stage and age of patients are crucial prognostic factors [5].

Tumour cells are characterised by increased glucose uptake. These cells utilise glucose mainly through glycolysis, which leads to the production of lactate. Tumour cell glycolysis is also being investigated as a potential target for cancer treatment and as a possible therapeutic target [6–8]. Increased glycolytic activity leads to increased chemical resistance in some malignant tumours, which provides a favourable environment for survival of tumour cells [9]. The specific mechanisms through which tumour cells enhance the glycolytic process in Ewing's sarcoma are not yet known. Thus, the present study attempted to investigate the role of glycolysis-related genes in Ewing's sarcoma.

With advancement of the bioinformatics field, increasing number of analyses based on this discipline are being utilised in clinical practice [10]. Application of bioinformatics analyses in immune cell infiltration for the treatment of cancer is also increasing, and immunotherapy seems to be the future therapeutic modality for cancers. Immune cells in the tumour microenvironment are the crucial components of the cancer development process that not only antagonise tumours but also promote tumour development, leading to tumour progression [11]. For many years, scholars have been aiming to develop an injectable vaccine, which could allow the human body to expand the immune response to tumour-specific T cells through active immunity, eventually achieving immunotherapy for cancer [12]. Immunotherapy is considered to play a crucial role in cancer treatment. Therefore, immune cells in Ewing's sarcoma were explored in the present study.

In this study, we aimed to identify glycolysis-related and immune-related prognostic biomarkers in Ewing's sarcoma through precise bioinformatics techniques in order to improve clinical prognosis, and we validated our analysis through the immunohistochemical analysis.

## MATERIALS AND METHODS

### Retrieval of Ewing's sarcoma data and glycolysis-related gene sets and preliminary processing of the data

Gene expression data and the corresponding clinical information data for Ewing's sarcoma were downloaded from the gene expression omnibus repository in the GSE17674 dataset [13]. Inclusion criteria were as follows: 1) samples with a diagnosis of Ewing's sarcoma; and 2) data with complete clinical information. A total of 13 skeletal muscle (vastus lateralis) samples were excluded, leaving only the skeletal muscle samples as normal controls. Gene sets related to glycolysis were downloaded from the gene set enrichment analysis (GSEA) database (http://www.gsea-msigdb.org/gsea/msigdb/search.jsp).

### Differential expression analysis and weighted gene co-expression network analysis

We used the programming language R (version 4.0.2) for all statistical analyses as well as for the visualisation of plots. To fully utilise the information in the expression matrix and identify key genes in Ewing's sarcoma, we used a combination of the differential expression analysis and weighted gene co-expression network analysis (WGCNA). Firstly, we performed the differential expression analysis of all genes by using the ggplot2, limma, and pheatmap packages, with cut-off values of |LogFC | > 2.5 and false discovery rate (FDR) < 0.01. The weighted co-expression network analysis is an advanced analytical method in bioinformatics for calculating gene-to-gene co-expression relationships by establishing and analysing a framework for weighted gene co-expression networks that allows complete utilisation of the information in the gene expression matrix [14]. A gene-to-gene similarity network was constructed, and the network modules were identified. Then, the relationship between the gene expression of cancer and normal groups was explored, and subsequently, the key genes in the modules were identified. Thereafter, the differentially expressed genes (DEGs) identified through the differential expression analysis were intersected, the modules with the highest tumour Pearson correlation coefficient were identified using the WGCNA, the glycolysis-related genes were downloaded from the GSEA database, and the intersected genes were saved for subsequent analysis.

### Gene ontology and Kyoto Encyclopedia of Genes and Genomes enrichment analyses

To further explore the cellular component, molecular function, biological process, and KEGG-enriched pathways of these genes, we ID-transformed these genes by using the colorspace, stringi, ggplot2, digest, and GOplot packages; analysed their gene ontology (GO) and Kyoto Encyclopedia of Genes and Genomes (KEGG) pathways; and subsequently visualised them.

### Univariate cox regression, regression, and multivariate cox regression analyses

To construct a prognostic model for Ewing's sarcoma, we used the univariate Cox regression analysis, least absolute shrinkage and selection operator (LASSO) regression analysis, and multivariate Cox regression analysis to screen genes from multiple angles and directions. First, we performed a single-factor Cox regression analysis of the gene-survival data from the previous intersection, and the cut-off value was set at P < 0.05. Secondly, to refine our prognostic model, we constructed a penalty function by using the LASSO regression analysis to obtain the optimal number of genes required for constructing the model. Finally, we used a multivariate Cox regression model for the multifactorial analysis of genes and survival status, with the cut-off value set at P < 0.05. Thus, we obtained a prognostic model, the gene name for which the prognostic model was constructed, and the risk-score for each sample.

### Survival analysis

We analysed the survival data from several perspectives. First, we established Kaplan–Meier survival curves for comparing the prognosis between high and low gene expression and survival based on the relationship between high and low gene expression and prognosis of the constructed model. Subsequently, based on the constructed model, we divided the patients into high- and low-risk groups on the basis of their risk values by using the median risk value as a criterion. Kaplan–Meier survival curves were constructed for comparing the prognosis and survival between high- and low-risk groups.

### Gene expression and principal component analysis of modelled genes

Expression calculations were performed for the gene expression of the constructed model based on the high and low risk of wind resistance, which was visualised as a violin plot. To verify the accuracy of the constructed model, we performed a compositional analysis of the high- and low-risk groups by using the ggplot2 package to investigate the differences between the two groups.

### Receiver operating characteristic diagnostic curve

To validate the accuracy of the constructed Ewing's sarcoma model, we calculated area under the curve (AUC) values for 1, 2, and 3 years based on the analysis of the SURVIVAL package, SURVEMINER, and TIMEROC package for receiver operating characteristic (ROC) diagnostic curves, respectively.

### Risk assessment

We ranked all the patients with Ewing's sarcoma in the descending order of their risk-score, and then determined the survival status of each patient. Finally, we plotted the gene expression by constructing the prognostic model for each patient.

### Assessment of accuracy of the prognostic model and prediction of the survival rate

To validate the accuracy of the constructed prognostic model of Ewing's sarcoma, we used the rms package to divide the patients into high- and low-risk groups based on their risk-score value and plotted calibration plots by predicting overall survival at 3 years. To predict survival in Ewing's sarcoma, we constructed line graphs for predicting the prognosis based on the risk of the genes used to construct the model.

### Analysis of the immune cell composition

CIBERSORT software was used to analyse the composition of immune cells for each patient. The CIBERSORT software [15] is an advanced software for evaluating the immune cell composition, and it can display the immune cell components in the expression matrix. The total value of the 22 immune cell compositions for each sample was 100%. We also constructed a heat map of immune cells to further understand the relationship between immune cells in Ewing's sarcoma. Finally, we preserved the statistically significant immune cell differences in Ewing's sarcoma (P < 0.05) for subsequent analyses.

### Immunohistochemistry

We used tumour sections and paracancerous tissue sections from patients with Ewing's sarcoma who underwent surgery at the First Clinical Affiliated Hospital of Guangxi Medical University for immunohistological studies, which were approved by the Ethics Department of the First Clinical Affiliated Hospital of Guangxi Medical University, and conformed to the World Medical Association Declaration of Helsinki. We performed the immunohistological analysis of each gene on a total of 24 pathological sections from 6 pairs (Ewing's sarcoma and paraneoplastic tissues). The immunohistochemical analysis of the genes, which were used to construct the model, was performed to determine their protein expression in cancerous and paraneoplastic tissues. Antibodies for the immunohistochemical analysis were purchased from the Bioss (www.bioss.com.cn, item numbers: bs-1625R, bs-4042R). Immunohistochemical staining of the fixed formalin solution, paraffin-embedded Ewing's sarcoma tissue, and paraneoplastic tissue was performed. After removal of paraffin, hydration, and sealing, we incubated the tissue specimens with antibodies overnight at 4° C, all at a dilution ratio of 1:500. We used inverted microscopy to observe differences in the protein expression of individual genes in Ewing's sarcoma tissue and paraneoplastic tissue. Subsequently, we used Image J software to count the positivity rate of specific regions in each immunohistochemical image. We imported the positivity rate results for each image into IBM SPSS Statistics 25 and performed a statistical analysis of the positivity rates by using the t test of the two-paired sample means. Finally, we visualised the results of positivity rates for these two genes by using GraphPad Prism 8.

## RESULTS

### Data download

The GSE17674 dataset comprised 62 samples, which included 44 samples of Ewing's sarcoma, 5 samples of skeletal muscle, and 13 samples of skeletal muscle (vastus lateralis). According to the inclusion criteria, Ewing's sarcoma with a diagnosis of Askin and PNET were excluded, and a total of 32 samples with a diagnosis of Ewing's sarcoma and 5 normal control samples were finally included for the subsequent analysis. A total of 13 glycolysis-related gene sets for 317 glycolysis-related genes were downloaded from the GSEA database.

### Differential expression analysis and WGCNA

**Figure 1:**
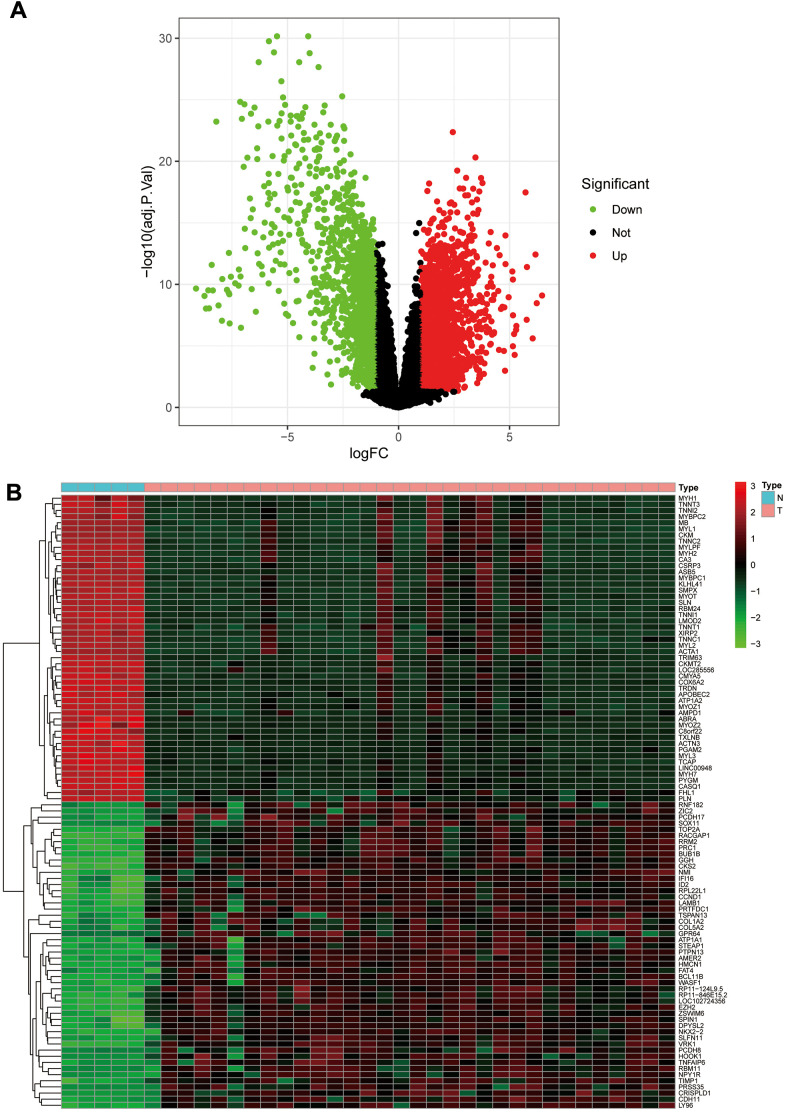
**The volcano plot and heat map of differentially expressed genes.** (**A**) The volcano plot; red dots are upregulated genes, green dots are downregulated genes, and black dots are nonsignificant differentially expressed genes. (**B**) The heat map; red rectangular blocks are highly expressed genes, and green rectangular blocks are poorly expressed genes.

**Figure 2:**
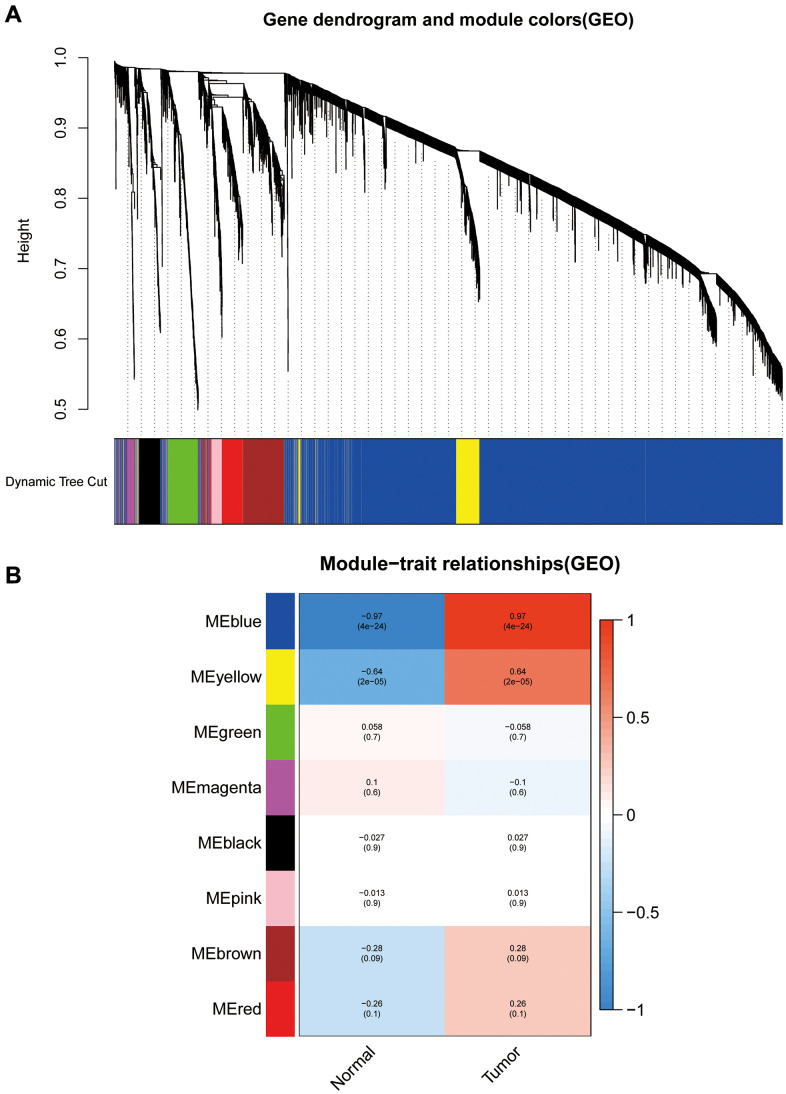
**Weighted gene co-expression network analysis dendrograms and correlation plots.** (**A**) The dendrogram. (**B**) The correlation high heat map of the modules.

**Figure 3:**
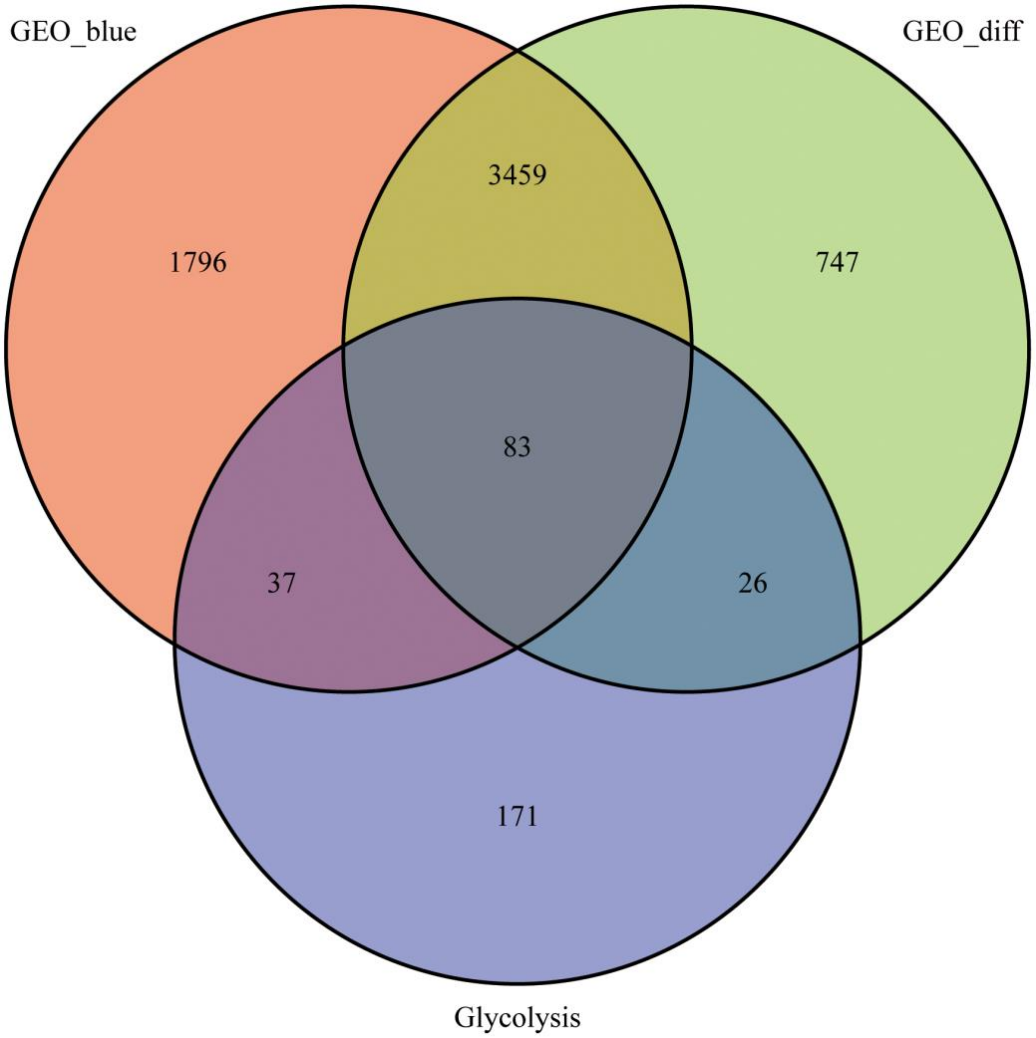
**Venn diagram.** 83 intersecting genes for the blue module, differentially expressed genes and glycolysis-related genes identified using the WGCNA.

We calculated DEGs from the expression matrices of 21,655 genes for 32 Ewing sarcomas and 5 normal controls, yielding 4314 significant DEGs according to pre-specified conditions. We visualised the DEGs as a volcano map (Figure 1A) and a heat map (Figure 1B). We have placed the details of the top 100 differentially expressed genes in Supplementary Table 1. Subsequently, we performed a complex WGCNA and obtained a dynamic shear tree (Figure 2A) and a significant disease-related difference module (Figure 2B). The MEblue module displayed the highest Person's correlation coefficient with Ewing's sarcoma (0.97). Therefore, we selected this gene module for further analysis. Subsequently, we constructed Venn plots of the MEblue module genes of DEGs, WGCNA, and glycolysis-related genes, which yielded 83 glycolysis-related genes with significant differences (Figure 3). We further filtered these genes.

### GO enrichment and KEGG pathway enrichment analyses

**Figure 4:**
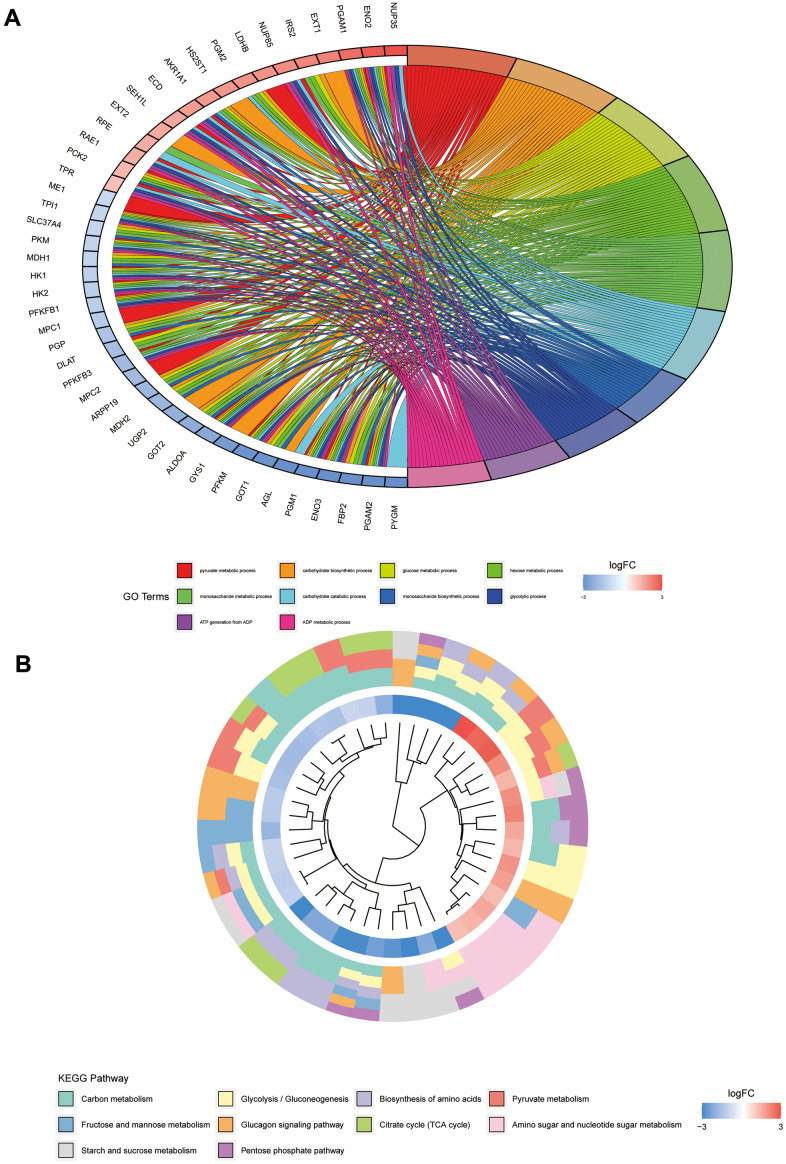
**GO and KEGG pathway analyses of glycolysis-related genes.** (**A**) Represents the GO pathway analysis; each colour represents a GO entry, and the top 10 GO entries are shown. (**B**) Represents the KEGG pathway analysis; each colour represents a pathway, and the innermost layer represents the size of the logFC value of the gene.

To further investigate the functions of these genes and the pathways involved, we performed the GO and KEGG enrichment analyses. Results of the GO enrichment analysis illustrated that GO is mainly enriched in the pyruvate metabolic process, carbohydrate biosynthetic process, glucose metabolic process, hexose metabolic process, monosaccharide metabolic process, and carbohydrate catabolic process (Figure 4A). The KEGG pathway analysis indicated significant enrichment of carbon metabolism, glycolysis/gluconeogenesis, biosynthesis of amino acids, pyruvate metabolism, glucagon signalling pathway, citrate cycle (TCA cycle), amino sugar and nucleotide sugar metabolism, starch and sucrose metabolism, and pentose phosphate pathway (Figure 4B).

### Univariate cox, LASSO, and multivariate cox regression analyses

**Figure 5:**
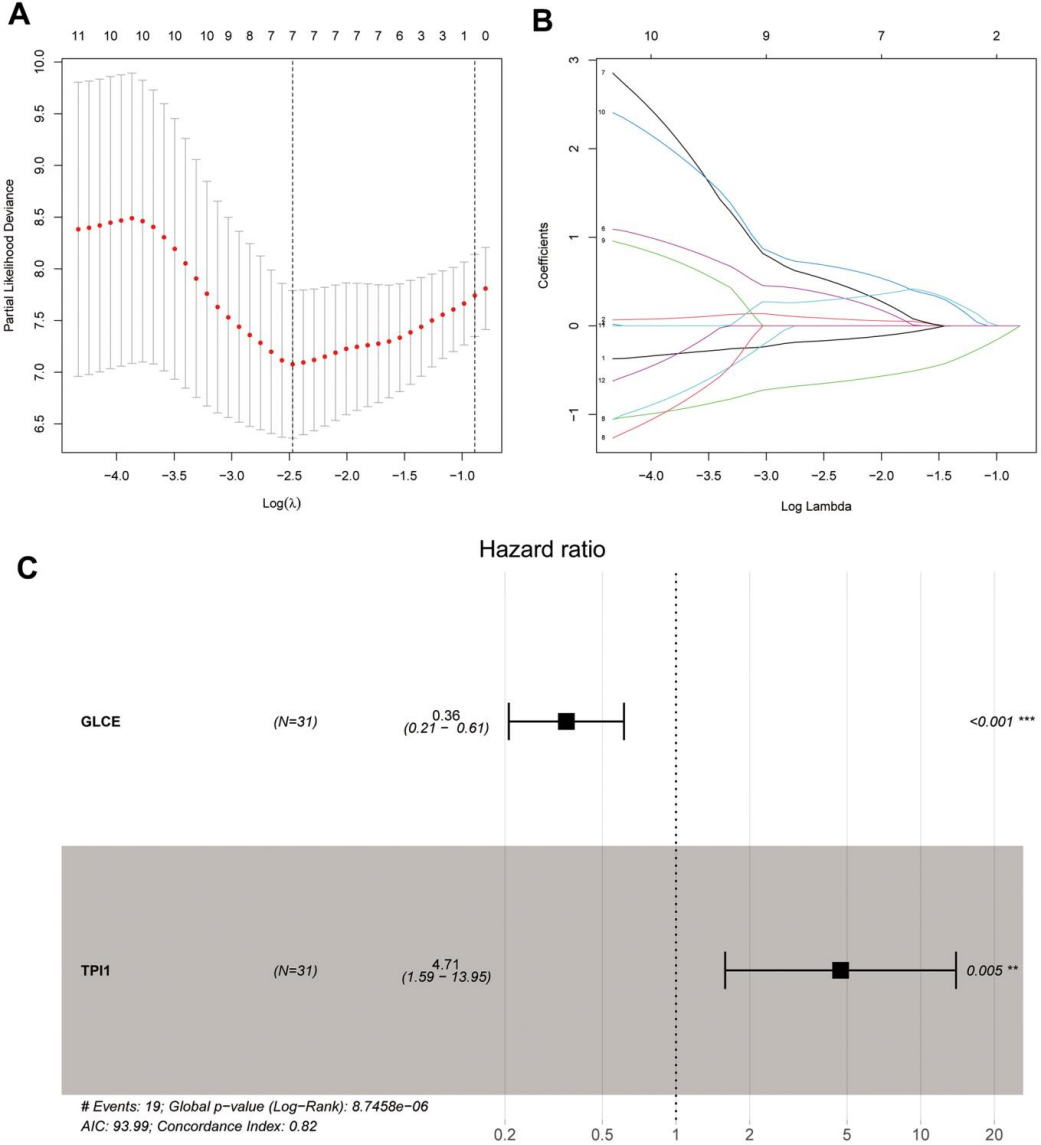
**Plots of LASSO regression and multifactor Cox analyses.** Plots (**A**, **B**) show the minimum penalty coefficients constructed using LASSO regression. Plots (**C**) indicates the multifactor Cox analysis, with P < 0.05 for both genes.

We performed univariate Cox, LASSO, and multivariate Cox regression analyses of the 83 significantly different glycolysis-related intersecting genes. After the univariate Cox regression analysis, the remaining 12 genes were found to meet our requirements. After a more complex and rigorous LASSO regression analysis (Figure 5A, 5B), only 7 genes met our requirements. Finally, we performed the multivariate Cox regression analysis, and only the remaining *GLCE* and *TPI1* genes were found to meet our requirements after screening (Figure 5C). We also obtained risk scores for each sample and divided all samples into the high-risk and low-risk groups.

### Survival analysis

**Figure 6:**
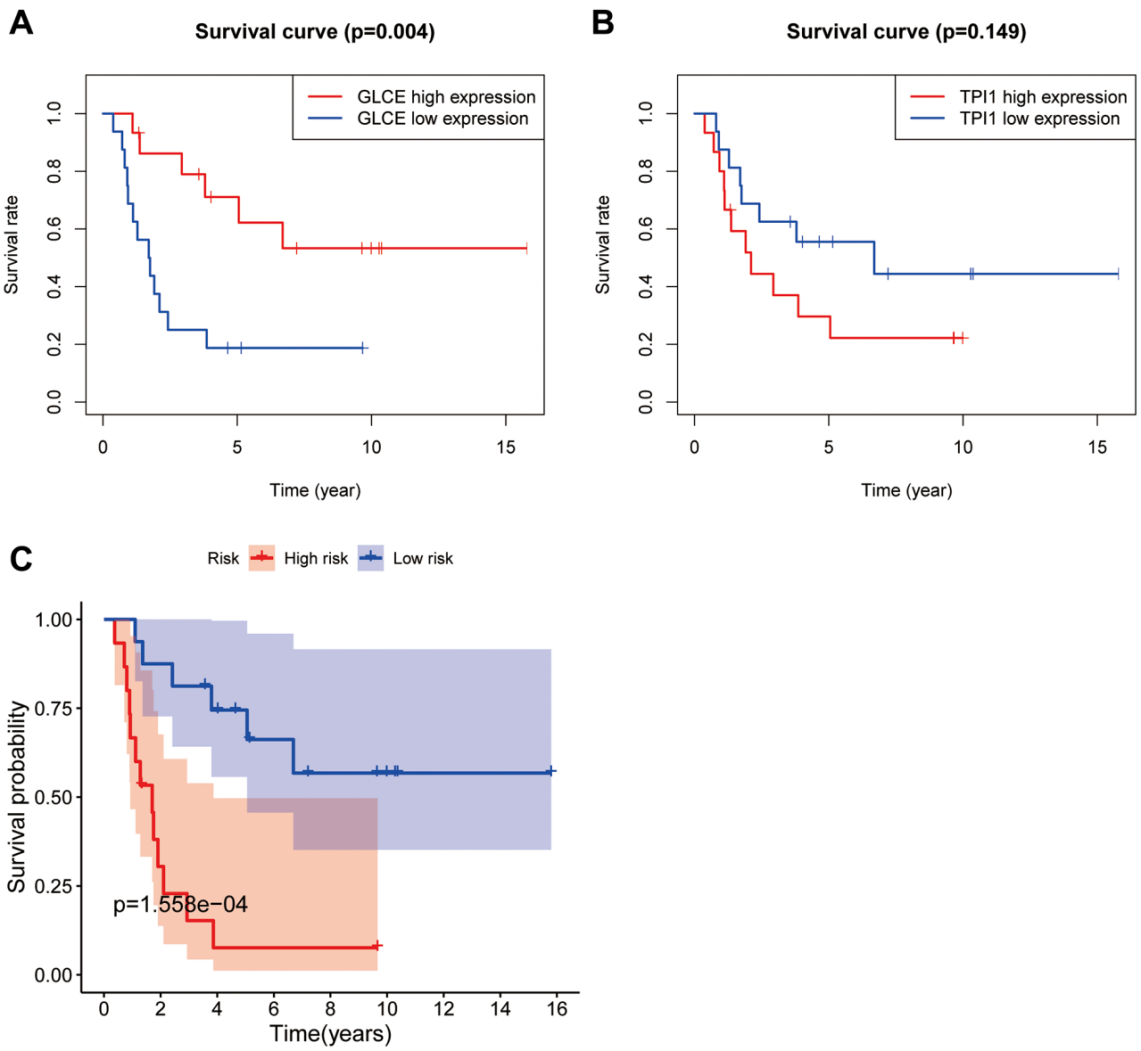
**Plots of survival analysis.** Plots (**A**, **B**) represent survival curves based on high and low expression of *GLCE* and *TPI1*. Plot (**C**) shows survival curves based on high and low risk of the model.

To utilise the survival information data, we analysed the survival information from two perspectives. As shown in Figure 6A, the patients with Ewing's sarcoma with high *GLCE* gene expression were found to have lower mortality and better prognosis compared with those having low GLCE gene expression, and the difference was found to be statistically significant (P < 0.01). However, high expression of the *TPI1* gene exhibited a worse prognosis compared with low expression (Figure 6B), and the difference was found to be statistically nonsignificant (P > 0.05). Kaplan–Meier survival curves plotted using the constructed prognostic model (Figure 6C) exhibited that the prognosis of patients in the high-risk group is much lower than in the low-risk group, and the difference was found to be statistically significant (P < 0.001).

### Model gene expression and principal component analysis

**Figure 7:**
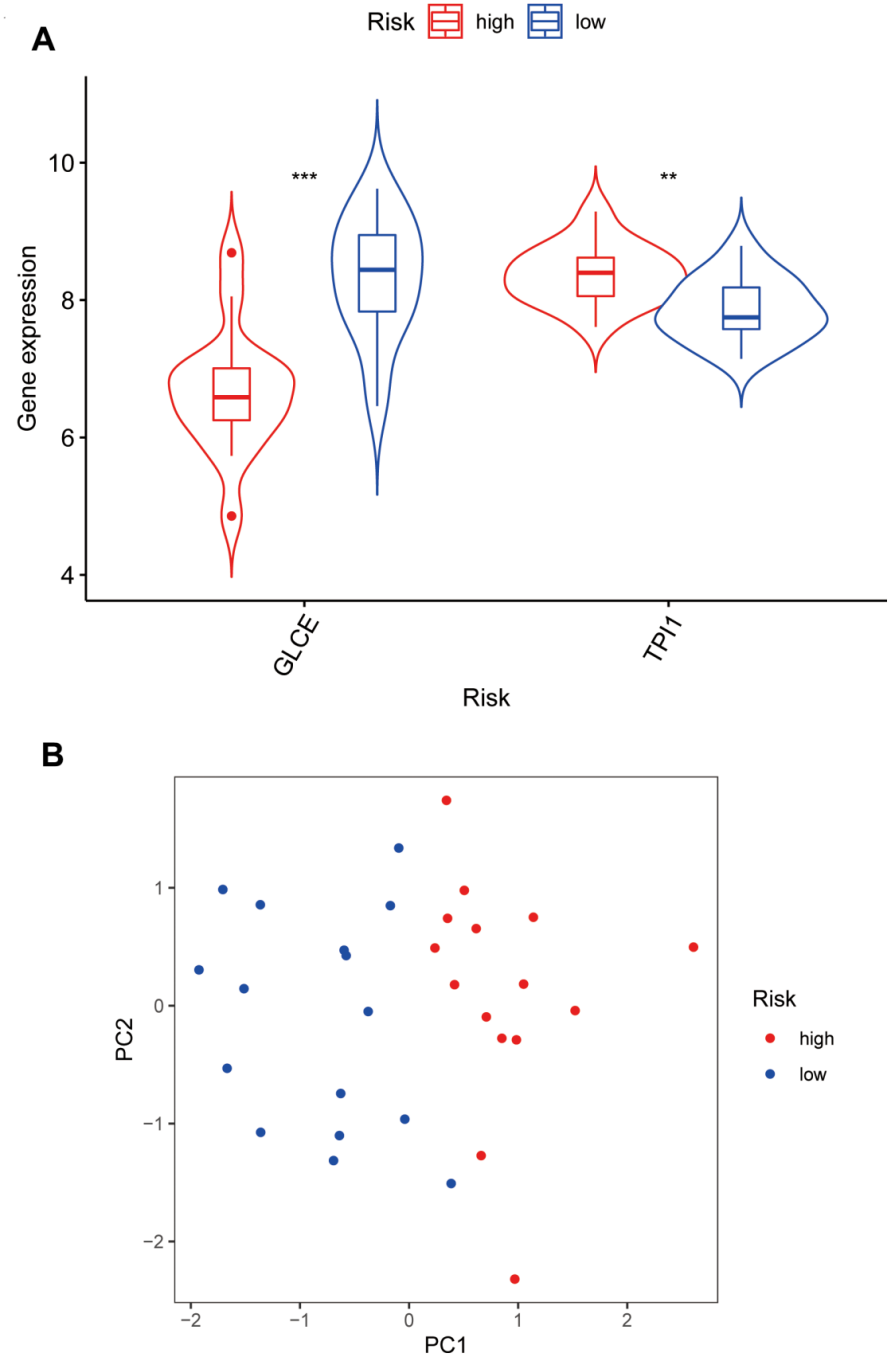
**Violin plots and principal component analysis plots of model gene expression based on high and low risk.** (**A**) The plot showing that *GLCE* expression values in the low-risk group are higher than those in the high-risk group; *TPI1* expression values in the high-risk group are higher than those in the low-risk group. (**B**) The principal component analysis plot; red points representing localisation of the high-risk group on the right side of the PC1 axis and that of the low-risk group on the left side of the PC1 axis.

We further analysed the two genes *GLCE* and *TPI1*, for which the model was constructed, to analyse the differential gene expression in the high- and low-risk groups. The expression of *GLCE* was found to be higher in the low-risk group than in the high-risk group, and the difference was statistically significant (P < 0.001) (Figure 7A). However, the expression of *TPI1* was found to be higher in the high-risk group than in the low-risk group, and the difference was statistically significant (P < 0.01). According to the constructed principal components analysis plot, the high-risk patients were concentrated on the right side of the PC1 axis, whereas the low-risk patients were located on the left side of this axis (Figure 7B).

### ROC diagnostic curve

**Figure 8:**
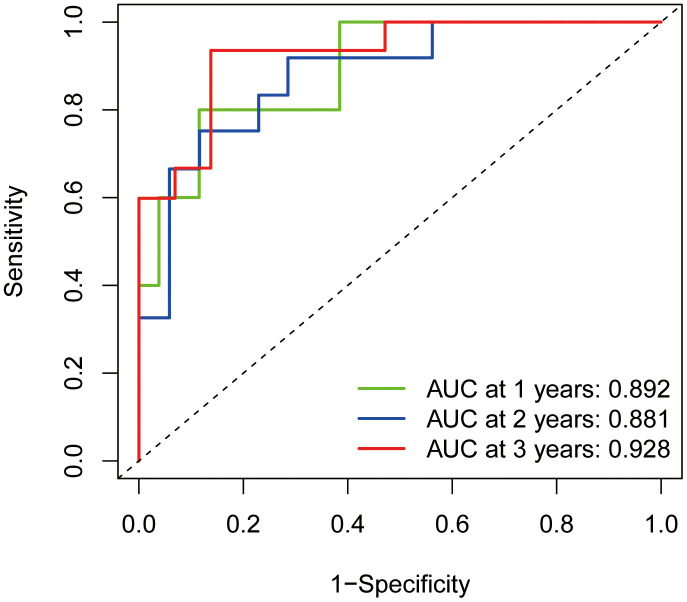
**ROC diagnostic curves.** The green line indicates the predicted 1-year survival rate; the blue line indicates the predicted 2-year survival rate; and the red line indicates the predicted 3-year survival rate.

The constructed ROC diagnostic curves indicated the AUC values for predicting survival to be much higher than 0.5; AUC values of 0.892, 0.881, and 0.928 for predicting 1-year survival, 2-year survival, and 3-year survival, respectively, were obtained (Figure 8). This finding also demonstrated the accuracy of the prognostic model constructed for Ewing's sarcoma.

### Risk assessment

**Figure 9:**
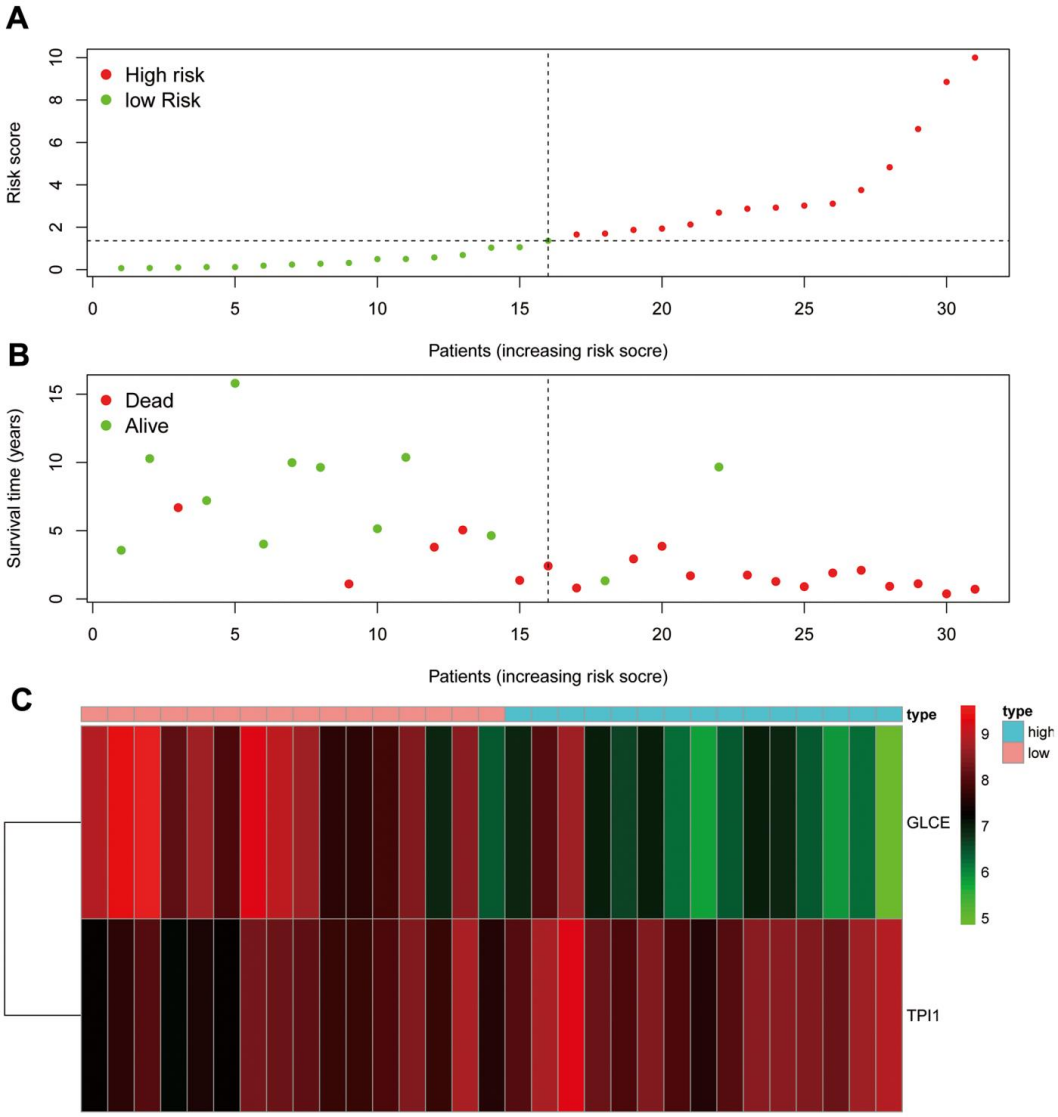
**Risk assessment graph.** (**A**) The risk score of the patients; (**B**) survival and death of the patients, and (**C**) the expression of the two genes in the high- and low-risk groups.

We ranked the patients in the descending order of their risk score (Figure 9A). In terms of patient survival, the number of deaths increased with the risk value (Figure 9B). The *GLCE* expression exhibited a decreasing trend, whereas *TPI1* expression exhibited an increasing trend from the high-risk group to the low-risk group (Figure 9C).

### Assessment of the prognostic model accuracy and prediction of the survival rate

**Figure 10:**
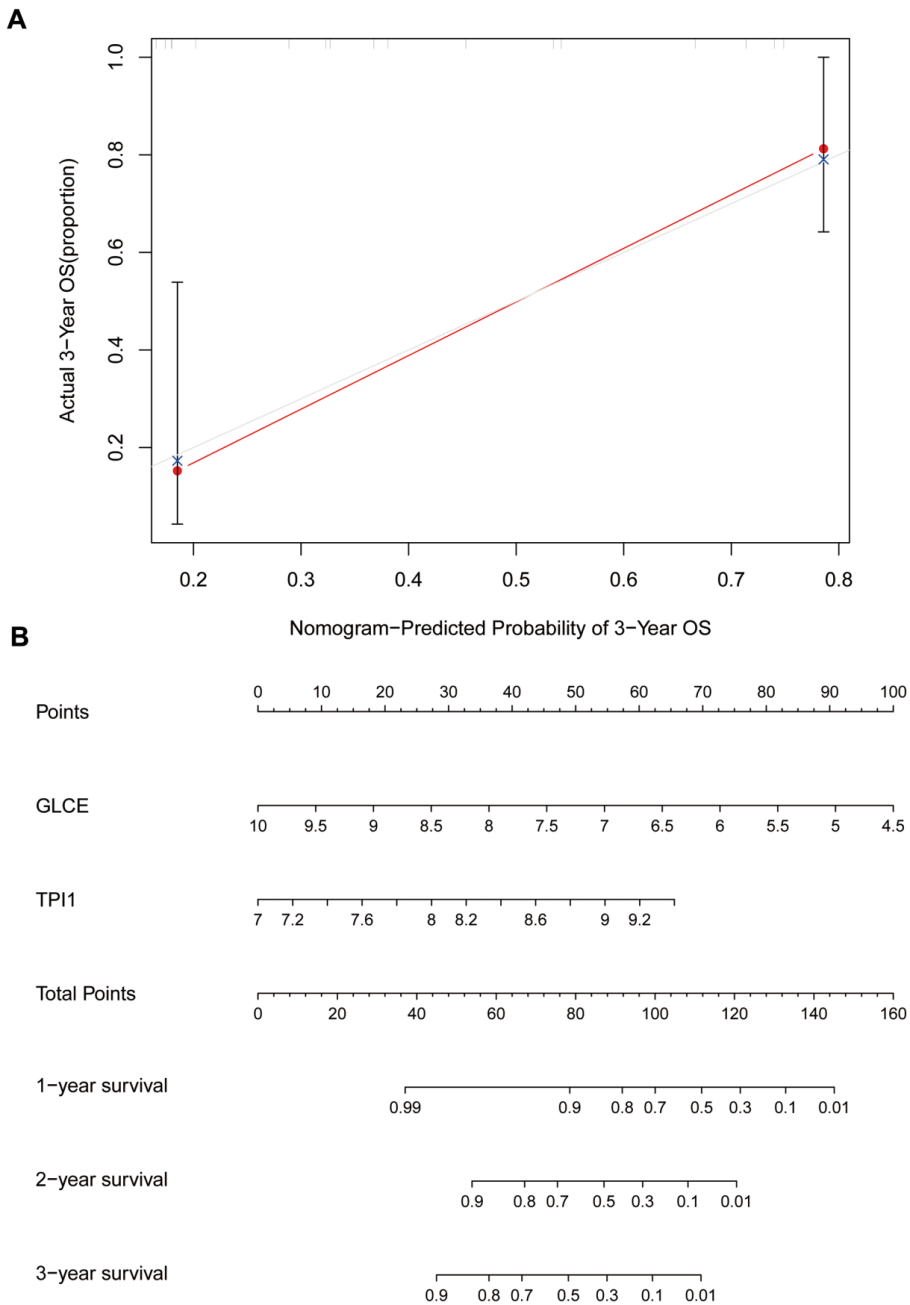
**Calibration and column line plots.** (**A**) The calibration plot; the red line segments denote the predicted line segments and the grey line segments denote the true-case segments. (**B**) The column line plots for the predicted prognosis.

We further constructed calibration plots to validate the rigour of the Ewing's sarcoma prognostic model (Figure 10A). The red line in Figure 10A almost coincides with the grey line, indicating that the difference between the predicted and the actual value is small. We also created a line graph (Figure 10B) for predicting survival based on *GLCE* and *TPI1* gene expression values, which could be used to predict 1-, 2-, and 3-year survival in patients with Ewing's sarcoma.

### Immune cell composition analysis

**Figure 11:**
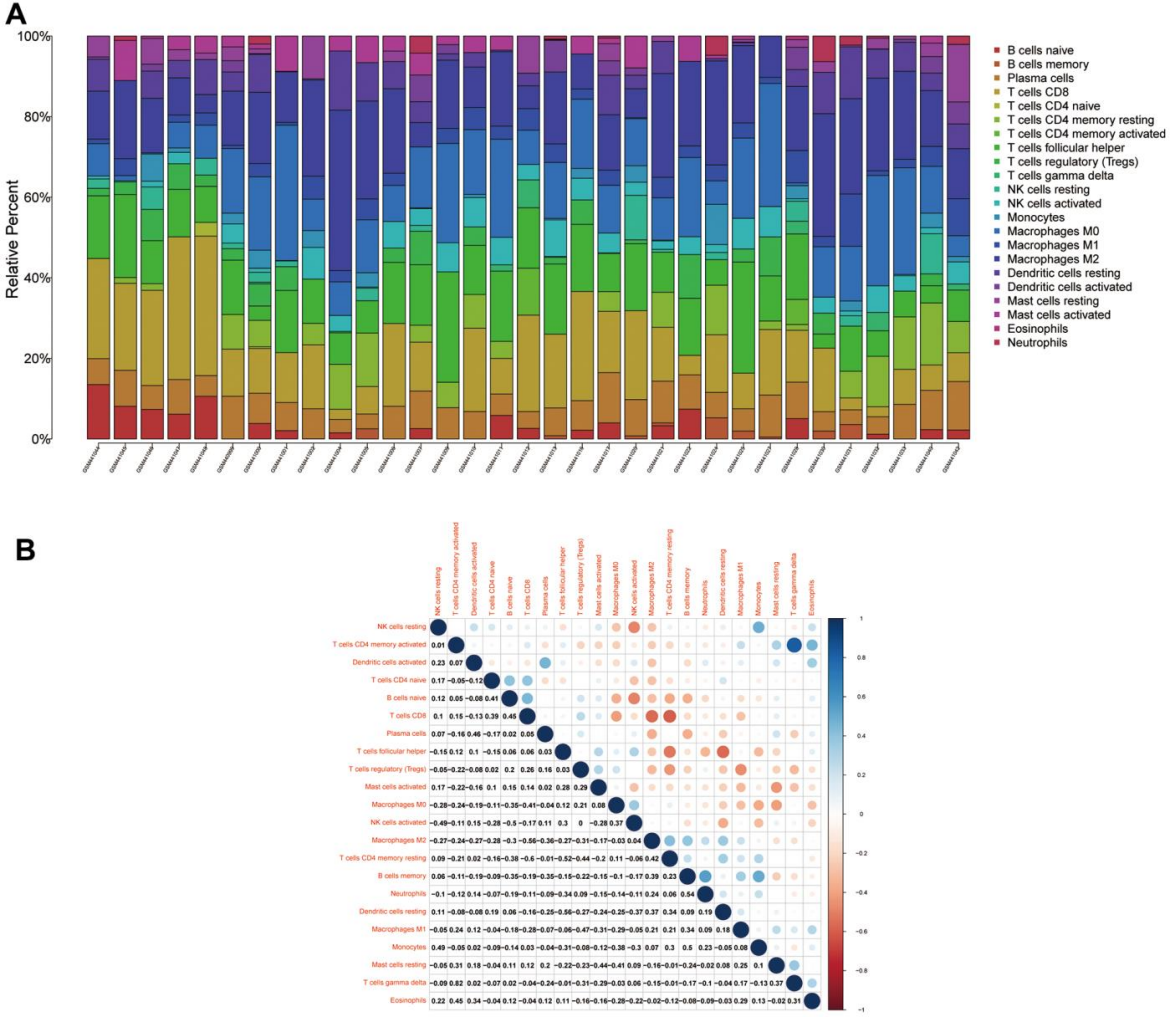
**Immune cell compositions and correlation heat map.** (**A**) Plot showing the composition of immune cells for each sample. (**B**) Plot showing the correlation heat map between each immune cells. Red indicates positivity correlation and blue indicates negative correlation.

**Figure 12:**
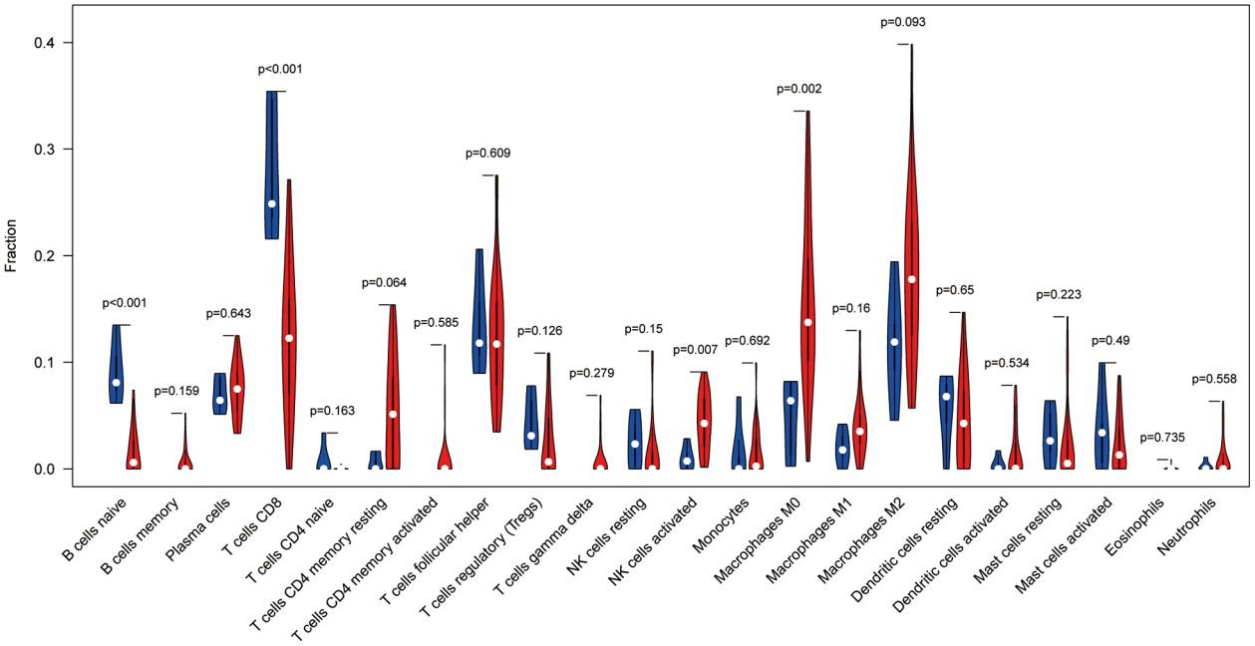
Immune cell violin plot showing that naïve B cells, CD8^+^ T cells, activated NK cells, and M0 macrophages are significant in Ewing's sarcoma (P < 0.05).

To analyse the relationship between Ewing's sarcoma and immune cells, we evaluated the immune cell composition of each sample by using CIBERSORT software. The immune cell components of each sample were visualised (Figure 11A), and the co-expression relationships between immune cells were also analysed (Figure 11B). Finally, we observed a significant association of naïve B cells, CD8 T cells, activated NK cells, and M0 macrophages with Ewing's sarcoma (P < 0.05) (Figure 12).

### Immunohistochemical analysis

**Figure 13:**
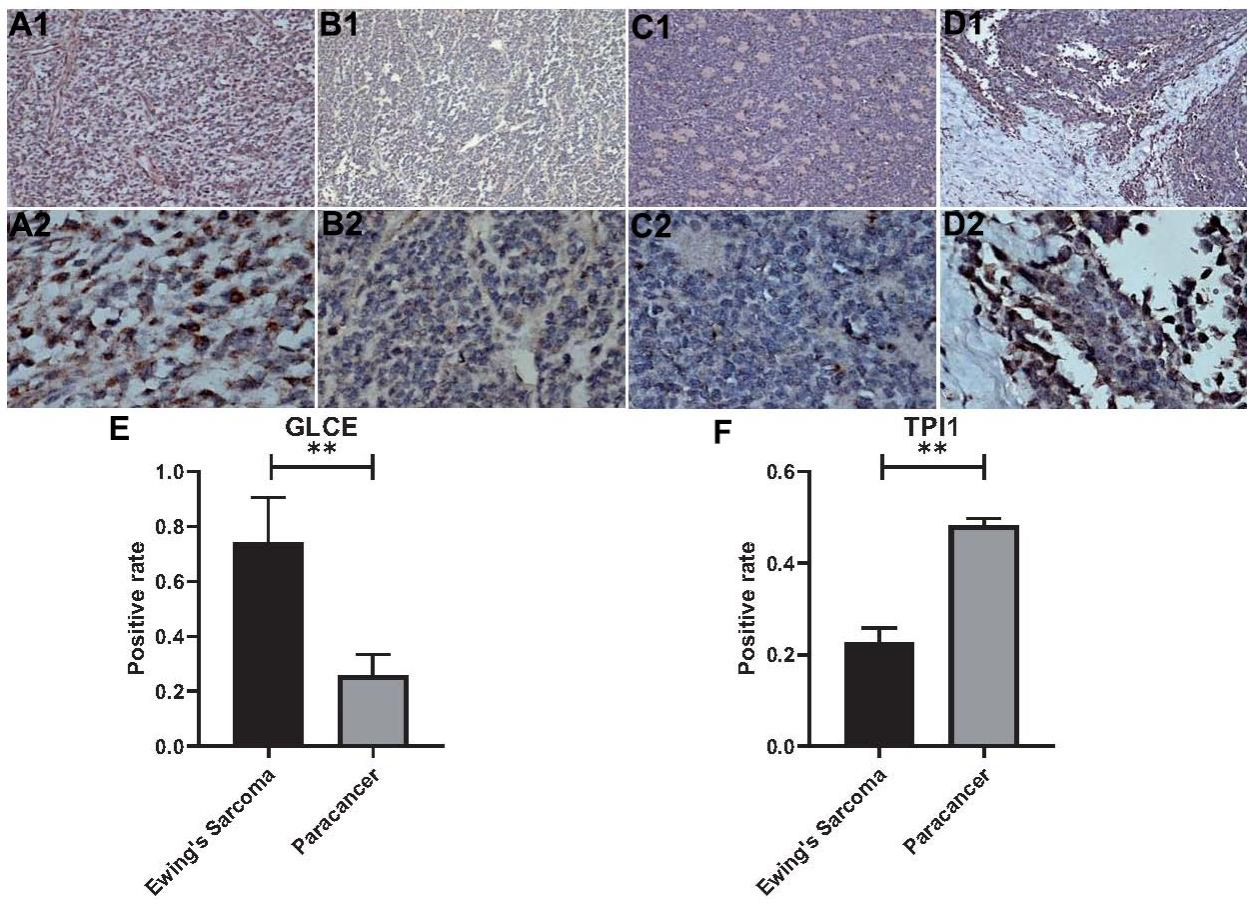
**Statistical analysis of immunohistochemistry and positivity rate.** (**A1**, **A2**) Show 100x magnification and 400x magnification of GLCE expression in cancerous tissue. (**B1**, **B2**) Show 100x magnification and 400x magnification of GLCE expression in paraneoplastic tissue. (**C1**, **C2**) Show 100x magnification and 400x magnification of TPI1 expression in cancerous tissue. (**D1**, **D2**) Show 100x magnification and 400x magnification of TPI1 expression in paraneoplastic tissue. (**E**) Indicates the statistical results of the positive rate of all immunohistochemical pictures of GLCE, “**” indicates P < 0.01. (**F**) Indicates the statistical results of immunohistochemical positivity rate for all TPI1, “**” indicates P < 0.01.

We performed the immunohistochemical staining analysis of six pairs (Ewing's sarcoma and paraneoplastic tissue) of a total of 24 pathological tissue sections for each gene. The antibodies used were specific for *GLCE* and *TPI1*, and brown-stained areas in the tissue sections are the result of a specific reaction between the antigen and antibody. As shown in Figure 13A1–13B2, GLCE expression was higher in immunohistochemically stained regions in Ewing's sarcoma than in paraneoplastic tissue. On the other hand, TPI1 was lower in immunohistochemically stained regions in 11. Immunohistochemical analysis of Ewing's sarcoma than in normal tissues (Figure 13C1–13D2). “Amend to”, as shown in Figure 13A1–13B2, GLCE expression was higher in immunohistochemically stained regions in Ewing's sarcoma than in paraneoplastic tissue. On the other hand, TPI1 was lower in immunohistochemically stained regions of Ewing's sarcoma than in normal tissues (Figure 13C1–13D2). In addition, we counted the positivity rate of a total of 24 specific immunohistochemical stains derived from *GLCE* and *TPI1*. Furthermore, we statistically analysed the positivity rate of Ewing's sarcoma staining and that of paraneoplastic tissue for both antibodies by using the t test for two-paired sample means in IBM SPSS Statistics 25. Results of the statistical analysis indicated the positivity rate of *GLCE* immunohistochemical-specific staining (Figure 13E) and implied that the mean number of samples positivity for *GLCE* in Ewing's sarcoma was higher than in the paraneoplastic tissue; the difference was found to be statistically significant. On the other hand, the rate of positivity staining for immunohistochemical-specific expression of *TPI1* was found to be higher in paraneoplastic tissues than in Ewing's sarcoma (Figure 13F), and the difference was found to be statistically significant. This finding was found to be consistent with those of our bioinformatics analysis, which further confirmed the accuracy of our analysis.

## DISCUSSION

In the present study, glycolysis-related genes with significant differences were found to be enriched mainly in the pyruvate metabolic process, carbohydrate biosynthetic process, glucose metabolic process, hexose metabolic process, monosaccharide metabolic process, and carbohydrate catabolic process. The KEGG pathway analysis indicated the significant enrichment of mainly the glycolysis/gluconeogenesis, biosynthesis of amino acids, pyruvate metabolism, glucagon signalling pathway, citrate acid cycle (TCA cycle), amino sugar and nucleotide sugar metabolism, starch and sucrose metabolism, and pentose phosphate pathway. Glycolysis plays a crucial role in cancer, and cancer cells derive energy in the form of lactic acid from glycolysis [16]. The metabolism of cancer cells differs from that of normal cells, and the metabolic rate of cancer cells is higher than that of normal cells, which enables cancer cells to maintain a sufficiently high proliferation rate to counteract cell death signals in the body. Additionally, glycolysis is enhanced in cancer cells [17]. Research on the cancer cell glycolysis, a pathway that may be integral to the development of cancer, has been conducted on a large scale [18–21]. In this study, we also observed that the glycolysis-related genes *GLCE* and *TPI1* can be used to construct a prognostic model of Ewing's sarcoma and that their high-risk group corresponds to a significantly poor survival status. This finding provides evidence for a role of glycolytic genes in Ewing's sarcoma.

Glucuronic acid epimerase (GLCE), is primarily associated with spherocytosis. A study conducted in 2011 on *GLCE* in cancer reported that the ectopic re-expression of *GLCE* exerts an antitumour effect and hence, it is a potential cancer suppressor gene [22]. In recent years, *GLCE* has been identified as one of the key enzymes involved in the biosynthesis of acetyl heparin sulfate, which plays a tumour-suppressive role in the pathogenesis of breast cancer [23]. A study on *GLCE* exhibited that a prognostic model consisting of 9 genes, including *GLCE*, displays shorter survival in the high-risk group than in the low-risk group [24]. This finding is concurrent with that of our study. The present study indicated that patients in the high *GLCE* expression group have a better prognosis compared with the low expression group, whereas the prognostic model of Ewing's sarcoma consisting of *GCLE* and *TPI1* demonstrated a worse prognosis in high-risk patients. *TPI1*, which is also a protein-coding gene, is primarily associated with triosephosphate isomerase deficiency and giardiasis. *TPI1* is located in the cytoplasmic and extracellular regions and can serve as a biomarker for the diagnosis of liver metastasis in colon cancer [25]. Recent studies have observed that *TPI1* expression is significantly upregulated in intrahepatic cholangiocarcinoma, and that the upregulated *TPI1* expression is strongly associated with high recurrences in patients with intrahepatic cholangiocarcinoma [26]. Studies on *TPI1* have reported that the gene plays a role not only in colon cancer but also in pancreatic cancer. Follia et al. reported that patients with enhanced glycolysis have earlier disease progression and poorer prognosis than other patients, leading to overexpression of *TPI1* [27]. This finding is consistent with that of our study. The present study indicated that a high expression of *TPI1* in Ewing's sarcoma has an overall poor prognosis compared with low *TPI1* expression. Here, the prognostic model for Ewing's sarcoma that comprised *TPI1* exhibited that the prognosis of patients in the high-risk group is much lower than that of patients in the low-risk group.

We not only established a prognostic model but also analysed immune cells associated with Ewing's sarcoma. Characteristics of B cells such as naïve B cells, the ease with which they can be activated, and their lifespan are likely to be the indispensable factors for the malignant transformation of chronic lymphocytic leukaemia [28]. CD8^+^ T cells are the immune cells that are generally preferred for the targeted cancer therapy. However, during cancer development, the tumour immune microenvironment causes immunosuppression, which results in adaptive immune resistance. Thus, CD8^+^ T cells play an antitumour role in cancer [29]. An increasing number of studies have shown that NK cells are capable of exhibiting cytotoxic activity against a wide range of tumour cells and that enhancing the antitumour immunity of NK cells is a vital approach to immunotherapy for cancer [30]. Stimulation of M0 macrophages with apoptotic SKOV3 cells enables these macrophages to allow tumour cell migration and proliferation [31]. The present study revealed that all four immune cells are strongly associated with Ewing's sarcoma, and our findings might provide a new therapeutic direction for Ewing's sarcoma with a poor prognosis.

In the present study, we screened the significant preliminary differences in glycolysis-related genes by combining the differential expression analysis with the WGCNA and then intersecting with glycolytic genes. Subsequently, we used univariate Cox, LASSO, and multifactorial Cox regression analyses of the genes, which were used to construct an accurate prognostic model for Ewing's sarcoma. To validate the accuracy of the model, we analysed patient survival information in relation to the model, gene expression based on high- and low-risk groups, principal components analysis, ROC diagnostic curves, risk assessment, calibration plots, and line graphs. We performed the immune cell evaluation of Ewing's sarcoma by using CIBERSORT software and observed that naïve B cells, CD8^+^ T cells, activated NK cells, and M0 macrophages are all strongly associated with the disease. Finally, we confirmed our analysis through the immunohistochemical analysis. The *GLCE* expression was significantly higher in Ewing's sarcoma than in paraneoplastic tissues, whereas the *TPI1* expression was significantly higher in paraneoplastic tissues than in Ewing's sarcoma, which is consistent with our bioinformatics analysis results.

Tumour cells are known to avoid death through immune evasion [32]. Interestingly, in our study, the expression profile of Ewing's sarcoma was compared with that of immune cells by using the bioinformatics algorithm, which indicated that naïve B cells, CD8^+^ T cells, activated NK cells, and M0 macrophages differ significantly in Ewing's sarcoma (P < 0.05). Therefore, we believe that the role played by these immune cells in the development of Ewing's sarcoma requires further investigation, which could provide a new guideline for immunotherapy of Ewing's sarcoma. We believe that in the upcoming 5 years, research on tumour immunity will be strengthened and provide novel and effective strategies for better treatment of tumours.

The present study has some limitations. First, the sample size was inadequate; we could not obtain a large sample size for the analysis. Second, we did not specifically analyse the typology of Ewing's sarcoma, despite the presence of various types of Ewing's sarcoma. Finally, we analysed only the survival information of Ewing's sarcoma and did not further analyse other clinical information.

## CONCLUSIONS

The prognostic model of Ewing's sarcoma constructed using *GCLE* and *TPI1* indicated that survival of the high-risk group is much lower than that of the low-risk group and that *GCLE* and *TPI1* can serve as prognostic biomarkers for Ewing's sarcoma.

### Ethical disclosure

This study has passed the ethical review of the First Clinical Affiliated Hospital of Guangxi Medical University.

### Data sharing statement

The datasets supporting the conclusions of this article are available in the GEO Datasets (https://www.ncbi.nlm.nih.gov/geo/query/acc.cgi?acc=GSE17674).

## Supplementary Material

Supplementary Table 1
