# Supplementary material for: Glycolysis- and immune-related novel prognostic biomarkers of Ewing's sarcoma: glucuronic acid epimerase and triosephosphate isomerase 1
Source: Aging (Albany NY). 2021 Jul 7;13(13):17516–35. doi: 10.18632/aging.203242 (PMC8312448; doi:10.18632/aging.203242)
Supplement: Supplementary Table 1 [file aging-13-203242-s001.docx]

**Supplementary Table 1. Graph of the top 100 differentially expressed genes.**

| id | logFC | AveExpr | t | P.Value | adj.P.Val | B |
| --- | --- | --- | --- | --- | --- | --- |
| FBP2 | -5.47298 | 4.616873 | -47.5432 | 4.69E-35 | 6.91E-31 | 69.04792 |
| FSD2 | -4.06709 | 4.509861 | -47.147 | 6.39E-35 | 6.91E-31 | 68.7687 |
| ASB2 | -5.83708 | 5.110332 | -45.4685 | 2.42E-34 | 1.75E-30 | 67.55387 |
| SMTNL1 | -5.60869 | 5.563512 | -42.6257 | 2.59E-33 | 1.40E-29 | 65.37129 |
| TRIM7 | -4.00389 | 4.387605 | -42.187 | 3.79E-33 | 1.64E-29 | 65.01949 |
| C3orf43 | -4.47197 | 3.865219 | -40.0428 | 2.56E-32 | 8.79E-29 | 63.23781 |
| ABRA | -6.29797 | 3.904218 | -39.9275 | 2.84E-32 | 8.79E-29 | 63.13898 |
| KLHL34 | -3.60125 | 3.866994 | -38.7906 | 8.16E-32 | 2.21E-28 | 62.14692 |
| AK056982 | -5.27588 | 4.403965 | -35.9586 | 1.29E-30 | 3.11E-27 | 59.52905 |
| GLCE | 3.852229 | 6.942541 | 7.365503 | 8.71E-09 | 7.53E-08 | 9.48536 |
| ASB10 | -2.5376 | 4.592866 | -33.1696 | 2.42E-29 | 5.25E-26 | 56.72306 |
| PPP1R3A | -5.20085 | 3.648265 | -32.8987 | 3.26E-29 | 6.42E-26 | 56.43729 |
| CASQ1 | -7.13099 | 4.614345 | -32.0784 | 8.12E-29 | 1.47E-25 | 55.55653 |
| MYL3 | -6.93871 | 5.736889 | -31.6021 | 1.39E-28 | 2.32E-25 | 55.03431 |
| DUSP13 | -5.10757 | 5.487376 | -31.4414 | 1.68E-28 | 2.59E-25 | 54.85623 |
| P2RY2 | -3.32622 | 4.644491 | -31.2844 | 2.01E-28 | 2.90E-25 | 54.68141 |
| MBNL1-AS1 | -4.19488 | 4.797317 | -30.9573 | 2.93E-28 | 3.97E-25 | 54.31415 |
| PGAM2 | -6.48438 | 4.657656 | -30.8412 | 3.36E-28 | 4.28E-25 | 54.18279 |
| SLC25A4 | -3.38973 | 5.982385 | -30.0407 | 8.65E-28 | 1.04E-24 | 53.26346 |
| PLIN4 | -4.30304 | 5.624071 | -29.8672 | 1.07E-27 | 1.21E-24 | 53.06088 |
| FBXO40 | -5.26635 | 4.794587 | -29.7903 | 1.17E-27 | 1.26E-24 | 52.97075 |
| ACTN3 | -6.64293 | 5.087765 | -29.692 | 1.32E-27 | 1.36E-24 | 52.8551 |
| VGLL2 | -4.85768 | 4.706944 | -29.3757 | 1.93E-27 | 1.90E-24 | 52.48045 |
| SRL | -4.54147 | 6.227302 | -29.245 | 2.27E-27 | 2.10E-24 | 52.32447 |
| MYH6 | -4.58843 | 4.577568 | -29.2227 | 2.33E-27 | 2.10E-24 | 52.29779 |
| DHRS7C | -4.35878 | 4.527821 | -28.8089 | 3.89E-27 | 3.37E-24 | 51.79886 |
| MYH7 | -7.05353 | 5.510574 | -28.743 | 4.22E-27 | 3.51E-24 | 51.71873 |
| IDI2 | -4.45883 | 5.175417 | -28.7124 | 4.38E-27 | 3.52E-24 | 51.68143 |
| RPL3L | -5.35836 | 5.477069 | -28.359 | 6.83E-27 | 5.28E-24 | 51.24806 |
| PYGM | -8.20688 | 5.603259 | -28.2354 | 7.98E-27 | 5.94E-24 | 51.09522 |
| TXLNB | -5.86246 | 3.818025 | -28.1923 | 8.43E-27 | 5.94E-24 | 51.04178 |
| ATP2A1 | -4.85632 | 4.680784 | -28.185 | 8.51E-27 | 5.94E-24 | 51.03278 |
| ADCK3 | -3.78527 | 6.048756 | -27.7105 | 1.56E-26 | 1.06E-23 | 50.43868 |
| LSMEM1 | -3.05378 | 4.414643 | -27.6802 | 1.62E-26 | 1.07E-23 | 50.40037 |
| C10orf71 | -5.45863 | 5.173685 | -27.5305 | 1.97E-26 | 1.25E-23 | 50.21071 |
| EPM2A | -2.47927 | 4.961373 | -27.386 | 2.38E-26 | 1.43E-23 | 50.02652 |
| LINC00948 | -6.31385 | 4.454623 | -27.3846 | 2.38E-26 | 1.43E-23 | 50.02482 |
| PLCD4 | -4.51491 | 4.449688 | -27.3194 | 2.59E-26 | 1.52E-23 | 49.94138 |
| NPY6R | -2.4385 | 4.196266 | -27.0339 | 3.77E-26 | 2.15E-23 | 49.5739 |
| EEF1A1 | 2.444127 | 10.87691 | 26.50049 | 7.66E-26 | 4.25E-23 | 48.87702 |
| SMYD1 | -4.26496 | 4.909913 | -26.3677 | 9.16E-26 | 4.96E-23 | 48.70134 |
| TMEM182 | -3.53779 | 4.042348 | -26.3073 | 9.94E-26 | 5.25E-23 | 48.6212 |
| LDB3 | -4.83266 | 5.756212 | -25.9647 | 1.58E-25 | 8.10E-23 | 48.16303 |
| PTGES3L | -3.14188 | 3.724432 | -25.9533 | 1.61E-25 | 8.10E-23 | 48.14774 |
| JPH2 | -2.76872 | 4.82119 | -25.9266 | 1.67E-25 | 8.21E-23 | 48.1118 |
| ITGB1BP2 | -3.70444 | 4.523624 | -25.6756 | 2.35E-25 | 1.13E-22 | 47.77195 |
| UCP3 | -2.82206 | 5.098799 | -25.5395 | 2.84E-25 | 1.34E-22 | 47.58627 |
| KLHL40 | -5.26789 | 4.678786 | -25.4859 | 3.06E-25 | 1.41E-22 | 47.51291 |
| MYOM3 | -4.06695 | 4.871111 | -25.3391 | 3.76E-25 | 1.70E-22 | 47.31113 |
| ATP1B4 | -4.25697 | 3.780849 | -25.2634 | 4.18E-25 | 1.85E-22 | 47.20675 |
| USP2 | -2.45275 | 4.627686 | -25.1317 | 5.03E-25 | 2.18E-22 | 47.02433 |
| RNF157 | -2.87569 | 4.628313 | -25.0859 | 5.36E-25 | 2.28E-22 | 46.96059 |
| NDRG2 | -3.46928 | 5.556778 | -24.8974 | 7.00E-25 | 2.91E-22 | 46.69746 |
| TMEM52 | -4.65323 | 4.425408 | -24.6328 | 1.02E-24 | 4.17E-22 | 46.32476 |
| LRRC39 | -4.61167 | 4.418763 | -24.5096 | 1.22E-24 | 4.88E-22 | 46.14991 |
| CTD-2083E4.7 | -3.66008 | 4.534784 | -24.0982 | 2.21E-24 | 8.70E-22 | 45.56006 |
| TCAP | -6.36667 | 6.353246 | -24.0789 | 2.27E-24 | 8.79E-22 | 45.5321 |
| SCN4A | -3.55146 | 4.964315 | -23.9718 | 2.66E-24 | 1.01E-21 | 45.37688 |
| C20orf166 | -3.77348 | 7.331836 | -23.8661 | 3.11E-24 | 1.15E-21 | 45.22309 |
| OBSCN | -3.68798 | 5.767861 | -23.8586 | 3.14E-24 | 1.15E-21 | 45.21215 |
| C10orf71-AS1 | -2.78997 | 4.161228 | -23.8379 | 3.24E-24 | 1.15E-21 | 45.1819 |
| ALPK3 | -4.32958 | 5.238099 | -23.8376 | 3.24E-24 | 1.15E-21 | 45.18143 |
| GMPR | -4.94669 | 4.882194 | -23.6031 | 4.59E-24 | 1.60E-21 | 44.83741 |
| TAL2 | -2.16101 | 4.656037 | -23.2422 | 7.87E-24 | 2.71E-21 | 44.30162 |
| LMOD3 | -5.13284 | 3.806024 | -23.0142 | 1.11E-23 | 3.76E-21 | 43.95914 |
| LOC100507537 | -5.67233 | 4.082255 | -23.0028 | 1.13E-23 | 3.77E-21 | 43.94197 |
| MYLK2 | -3.57394 | 6.175831 | -22.9451 | 1.24E-23 | 4.05E-21 | 43.85476 |
| CLIC5 | -3.58768 | 4.83586 | -22.8314 | 1.47E-23 | 4.75E-21 | 43.68221 |
| PABPC3 | 3.462576 | 11.14553 | 22.80718 | 1.53E-23 | 4.86E-21 | 43.64545 |
| COX6A2 | -6.80548 | 5.525163 | -22.772 | 1.61E-23 | 5.05E-21 | 43.59189 |
| ADSSL1 | -4.1996 | 5.002688 | -22.6903 | 1.83E-23 | 5.65E-21 | 43.46717 |
| TRDN | -6.36056 | 4.18458 | -22.579 | 2.17E-23 | 6.61E-21 | 43.29659 |
| RNF123 | -2.46272 | 6.584551 | -22.4053 | 2.84E-23 | 8.53E-21 | 43.02887 |
| MYO18B | -5.16633 | 4.524144 | -22.3878 | 2.92E-23 | 8.65E-21 | 43.00176 |
| TRIM54 | -3.67741 | 5.262529 | -22.2567 | 3.58E-23 | 1.05E-20 | 42.79827 |
| ACHE | -2.64173 | 4.636252 | -22.0534 | 4.93E-23 | 1.42E-20 | 42.48065 |
| SYPL2 | -4.47175 | 5.603569 | -21.9931 | 5.42E-23 | 1.54E-20 | 42.38583 |
| FABP3 | -3.99774 | 4.97474 | -21.6533 | 9.31E-23 | 2.62E-20 | 41.84748 |
| C8orf22 | -6.97297 | 4.577745 | -21.6054 | 1.00E-22 | 2.79E-20 | 41.77101 |
| RTN2 | -4.5908 | 6.17338 | -21.435 | 1.32E-22 | 3.62E-20 | 41.49757 |
| SLC25A34 | -2.81413 | 5.160386 | -21.3785 | 1.45E-22 | 3.92E-20 | 41.40642 |
| LRRC20 | -3.69203 | 6.204345 | -21.3309 | 1.57E-22 | 4.19E-20 | 41.32948 |
| KLHL31 | -3.20806 | 4.28501 | -21.3187 | 1.60E-22 | 4.22E-20 | 41.30973 |
| PRKAG3 | -3.41855 | 5.047724 | -21.1913 | 1.97E-22 | 5.13E-20 | 41.10303 |
| DYNLL1 | 2.642916 | 10.41819 | 21.11999 | 2.21E-22 | 5.69E-20 | 40.98689 |
| KIF1C | -2.4846 | 5.957568 | -21.0046 | 2.67E-22 | 6.80E-20 | 40.79804 |
| FEM1A | -2.76987 | 6.242554 | -20.9876 | 2.74E-22 | 6.91E-20 | 40.77017 |
| CTNNA3 | -1.98885 | 3.872748 | -20.8908 | 3.22E-22 | 8.01E-20 | 40.61107 |
| CAMK2B | -3.46 | 5.176711 | -20.8427 | 3.49E-22 | 8.58E-20 | 40.53169 |
| MSS51 | -4.01339 | 5.236545 | -20.7608 | 3.99E-22 | 9.71E-20 | 40.39619 |
| HHATL | -5.84814 | 5.033682 | -20.7083 | 4.36E-22 | 1.05E-19 | 40.30911 |
| TPI1 | -2.56886 | 8.229961 | -4.77092 | 2.80E-05 | 0.000101 | 1.437888 |
| NRAP | -5.4115 | 4.517679 | -20.6913 | 4.48E-22 | 1.07E-19 | 40.28088 |
| PCNT | -2.13011 | 5.826158 | -20.6337 | 4.93E-22 | 1.16E-19 | 40.18505 |
| TPM3 | -3.06262 | 5.890192 | -20.4535 | 6.67E-22 | 1.55E-19 | 39.88356 |
| CAV3 | -3.72999 | 6.125342 | -20.3979 | 7.33E-22 | 1.69E-19 | 39.79009 |
| ASB8 | -2.75255 | 6.551224 | -20.3687 | 7.70E-22 | 1.75E-19 | 39.74096 |
| ENO3 | -4.8443 | 4.922927 | -20.2526 | 9.37E-22 | 2.11E-19 | 39.54488 |
| CACNA1S | -4.80801 | 4.917678 | -20.2284 | 9.76E-22 | 2.18E-19 | 39.50379 |
